# Management of Acral and Mucosal Melanoma: Medical Oncology Perspective

**DOI:** 10.1093/oncolo/oyac091

**Published:** 2022-05-30

**Authors:** Seungyeon Jung, Douglas B Johnson

**Affiliations:** Vanderbilt University School of Medicine, Nashville, TN, USA; Department of Medicine, Vanderbilt University Medical Center and Vanderbilt Ingram Cancer Center, Nashville TN, USA

**Keywords:** mucosal melanoma, acral melanoma, targeted therapy, immunotherapy, PD-1, CTLA-4, PD-L1, *KIT*, BRAF

## Abstract

Acral and mucosal melanomas (MM) are rare subtypes of melanoma that are biologically and clinically distinct from cutaneous melanoma. Despite the progress in the treatment of cutaneous melanomas with the development of targeted and immune therapies, the therapeutic options for these less common subtypes remain limited. Difficulties in early diagnosis, the aggressive nature of the disease, and the frequently occult sites of origin have also contributed to the poor prognosis associated with acral and MM, with substantially worse long-term prognosis. The rarity of these subtypes has posed significant barriers to better understanding their biological features and investigating novel therapies. Consequently, establishing standardized treatment guidelines has been a challenge. In this review, we provide a brief overview of the current knowledge regarding acral and MM, focusing on their epidemiology, genetic backgrounds, and unique clinical characteristics. Further discussion centers around the management of primary and advanced disease and the role of emerging targeted and immune therapies for these subtypes, specifically focusing on issues relevant to medical oncologists.

## Teaser

This review provides an overview of acral and mucosal melanomas and highlights features that distinguish these rare subtypes from cutaneous melanoma. The article examines the efficacy and safety of current management strategies for localized and advanced disease in these subtypes and considers future directions for systemic therapy.

**Implications for Practice**

Acral and mucosal melanoma are uncommon, but still seen in clinical practice. This article reviews the unique surgical, adjuvant, and systemic therapy considerations of these subtypes.

## Introduction

Over the past decade, treatment for advanced cutaneous melanoma has advanced tremendously with the advent of effective targeted and immune therapies. Five-year median survival has improved from <10% historically to now more than 50% for patients treated with combination immunotherapy (1). Although the most common site of melanoma is sun-exposed skin (either with chronic or intermittent sun exposure), other anatomic regions may also have primary melanomas, including acral (palms, soles, nailbeds) and mucosal surfaces (gastrointestinal, genitourinary, sinonasal), and eye. While these less common sites have clearly benefited to some extent from novel melanoma therapies, their efficacy appears to be lower, and outcomes have lagged behind cutaneous melanoma. In this review, we will provide an overview of acral and mucosal melanomas (MM) and highlight features that distinguish these rare subtypes from cutaneous melanoma. We will then examine the efficacy and safety of current management strategies for localized and advanced disease in these subtypes and discuss future directions for systemic therapy.

## Acral Melanomas

### Background

Acral melanoma (AM) is a subtype of melanoma arising from the palms, soles, or nail apparatus. Although AM accounts for only 4%-6% of melanomas in Caucasians, it is the most common subtype of melanoma in Asians and blacks (2,3). Specifically, AMs make up a larger proportion of melanomas in blacks, Asians, and Hispanics due to the low incidence of UV-mediated melanomas in these ethnic groups (3,4). Patients with AM often have a worse prognosis compared with patients with cutaneous malignant melanoma. The 5-year overall survival (OS) for AM is inferior to that of cutaneous melanomas overall (80.3% and 91.3%, respectively; *P* < .001) (5).

Of note, non-Hispanic whites with AM have the highest 5-year survival rate, followed by blacks, Hispanic whites, and Asian/Pacific Islanders (82.5%, 77.2%, 72.8%, and 70.2%, respectively) (5). Delays in early diagnosis and advanced disease at presentation are thought to contribute to the poor prognosis associated with AM; as over two-thirds of AMs are diagnosed at stage II or above (compared with approximately one-third of cutaneous melanomas) (6). Early detection is complicated by difficulty in distinguishing early malignant melanoma from benign melanocytic nevus, the rarity of the tumor, and the often occult sites of origin (2). Moreover, the pigmentation of lesions of AM can follow the skin markings of the palms and soles, concealing the lesion and further hindering early diagnosis. There have also been numerous cases of AM being mistaken for more common conditions such as fungal infections and non-healing traumatic wounds (2). In recent years, dermoscopy has improved the diagnostic accuracy and early detection of AM (7).

### Management of Primary Disease and Role of Adjuvant Therapy

The standard management for primary AM is wide local excision. Given the location of AM, amputation is often needed (for subungual melanomas) or involvement of plastic surgery (palms/soles), which may lead to prolonged recovery periods, particularly for the lesions on the sole. However, for very early stage subungual melanomas, a retrospective analysis of 67 cases showed that nonamputative conservative treatment followed by a full-thickness skin graft is an effective and safe alternative to amputation; other retrospective studies have suggested this approach may be viable (8,9). However, subungual melanomas usually require amputation to achieve adequate margins, and sentinel lymph node biopsies (as with cutaneous melanomas) are recommended for AM. The role of adjuvant therapy in patients with localized AM following surgical excision remains unclear. Interferon alpha had been historically used as adjuvant therapy in patients with high-risk resected melanoma (stages IIb—III). Several randomized trials have been conducted that showed significant reduction in risk of melanoma recurrence in patients treated with IFNs (10,11); however, they did not consistently demonstrate an improvement in overall survival or benefit for patients with AM specifically. A phase II trial of 1 month versus 1 year of adjuvant high-dose interferon alpha-2b in high-risk patients with AMshowed no difference in relapse free survival (12). With the advent of targeted therapies and immunotherapy, there has been a shift towards these therapies for adjuvant therapy, which will be discussed later in this review.

## Mucosal Melanoma

### Background

Mucosal melanoma represents a rare subtype of melanoma, accounting for approximately 0.8%-3.7% of all melanomas in Caucasians (4,13,14). It can arise from any mucosal epithelium within the body, including epithelium from the lower GI tract (26.5%), nasal cavity and paranasal sinuses (23%), gynecological sites (22.5%), oral cavity (15%), urological sites (5%), upper GI tract (5%), and other (eg, conjunctiva, 3%) (15). While the incidence of cutaneous melanomas has increased by ~1.4% every year, the incidence of MM has remained stable (16). Mucosal melanoma is one of the most aggressive subtypes of melanoma and carries a significantly worse prognosis than cutaneous melanoma with a 5-year OS of 14% (17). Mucosal melanoma tends to present in older populations compared to CM (median age of diagnosis 70 vs. 55 years) (18). Gender disparities within MM exist with the incidence being 2 × higher in females, largely due to development of the disease in the genital tract (18). Racial disparities in incidence rates also exist. The incidence of MM is higher in Caucasians than other racial groups; however, MM accounts for a lower proportion of melanomas in Caucasians than non-White groups due to the higher incidence of cutaneous melanomas in Caucasians (19). Finally, MM differs by race/ethnicity with regard to anatomic site—non-Hispanic whites have the highest proportion of genitourinary MM, while Asian/Pacific Islanders most commonly have anorectal MM (20). Head and neck tumors are most frequently seen in Hispanics (20).

The etiology of MM remains unknown and modifiable risk factors have yet to be identified. Similar to AMs, sun exposure is not a risk factor for MM, leading to different mutational landscapes. Timely diagnosis is a challenge in MM (as in AM)—patients are more likely to present with symptoms and more advanced disease compared with patients with cutaneous melanoma. The rarity of this subtype and the occult site of origin contribute to difficulties in diagnosis.

### Management of Primary Disease and Role of Adjuvant Therapy

Complete surgical excision is the primary therapeutic strategy for MM. Obtaining negative pathological margins can be a challenge due to constraints depending on anatomic site and the lentiginous, multifocal pattern of growth. Even with negative margins, local recurrence is common, as are distant metastatic recurrences (21,22). Moreover, there can be considerable morbidity associated with extensive surgery due to the anatomic sites involved. Given this morbidity, the role of neoadjuvant therapy (discussed in Targeted and Immune Therapy sections, below) is being increasingly used in the clinic, although the clinical utility of this approach has not been systematically defined. The role of adjuvant therapy following surgical excision also remains unclear. Radiotherapy has been shown to improve locoregional control in several studies, but its impact on overall survival has not been determined (23-25). In cases of anorectal melanoma, sphincter-sparing local excision followed by radiation therapy may be an effective approach. In a retrospective study of 54 patients over 20 years, this approach achieved local control in 82% of cases and avoided the need for abdominoperineal resection and resultant colostomies (23). With regards to the role of adjuvant chemotherapy in the setting of MM, there has only been one randomized trial conducted. A randomized phase II study of 189 patients conducted in China demonstrated the superiority of temozolomide plus cisplatin in increasing OS (*P* < .01) and relapse-free survival (RFS) (*P* < .001) compared with high-dose interferon alfa-2b (HDI) and observation (26). Median RFS was 9.4 months in the HDI group and 20.8 months in the temozolomide plus cisplatin group, while median OS was 40.4 and 48.7 months, respectively (26). A phase III trial was then conducted comparing temozolomide plus cisplatin to HDI in 204 patients with resected MM. Median RFS was 15.5 months (95% CI 11.4-19.7) in the chemotherapy group compared with 9.5 months (95% CI 7.5-11.7) in the HDI group (*P* < .001). There was no statistically significant difference in median OS for chemotherapy versus HDI (41.2, 35.7 months; *P* = .083) (27). This regimen has not had widespread adoption given its toxicities and questions as to whether the results generalize to a Western patient population since these trials were conducted exclusively in Chinese patients. The authors note how the toxicities in the temozolomide plus cisplatin group differed from toxicities reported in Caucasians, suggesting that these regimens may need to be dosed with consideration to ethnicities (26). The introduction of immunotherapies and targeted therapies has led to a shift in the systemic treatment of MM, in the adjuvant setting and for advanced disease.

### Adjuvant and Neoadjuvant Immunotherapy Considerations

Adjuvant therapy with checkpoint inhibitors is currently approved for patients at high risk of recurrence following surgery. Unfortunately, there is very limited data investigating adjuvant immunotherapy in the setting of MM and AM due to the rarity of these subtypes and exclusion from trials. For instance, a phase III study of adjuvant pembrolizumab in high-risk stage III melanoma demonstrated significantly prolonged RFS; however, only patients with cutaneous melanoma (including acral) were included (28). Most of the data are extrapolated from smaller subgroup analysis. One phase III trial compared adjuvant nivolumab versus ipilimumab in 906 patients with resected stage IIIb-C and stage IV melanoma (Checkmate 238), of which 29 had MM and 34 had AM (29). At 4-year follow-up, nivolumab showed sustained RFS benefit compared with ipilimumab; however, in subgroup analysis of MM and AM, there was no statistically survival benefit associated with either agent (MM: hazard ratio, 1.71; 95% CI 0.68-4.29; AM: hazard ratio, 1.04; 95% CI 0.49-2.22) which may simply reflect low patient numbers (29). In a retrospective study, the efficacy of adjuvant PD-1 inhibitors in patients with CM was compared with patients with AM in the Asian population (30). Adjuvant anti-PD-1 treatment led to a better prognosis than HDI in patients with CM, but not in those with AM (30). In patients with CM, 12-month RFS rate (PD-1 inhibitor 77.8% vs. HDI 41.7%; 95% CI, 0.097-0.832; *P* = .014) was significantly higher in the PD-1 inhibitor group than in the HDI group, whereas in patients with AM, the 12-month RFS rate (PD-1 inhibitor 50.0% vs. HDI 61.6%; 95% CI, 0.567 to 4.499; *P* = .360) had no significant differences (30). One possible explanation for why adjuvant PD-1 inhibitor therapy is less efficacious in AM is due to its lower tumor mutational burden—higher tumor mutational burden has been associated with longer survival after ICI treatment (31). The mutational burden in AM is 20%-25% that of CM (32). Moreover, CM has significantly higher PD-L1 expression compared to AM (37.5% vs. 6.8%) and higher PD-L1 expression has also been shown to correlate with the efficacy of ICI therapy (33). It is also possible that these findings simply represent post-hoc comparisons with small numbers observed by chance alone. Although there remains a lack of data regarding the efficacy and safety of immunotherapy in these subtypes, patients at high risk of recurrence following excision of their primary acral or mucosal tumor can be considered for adjuvant immunotherapy (AJCC stage IIB/C or stage III). The efficacy of anti-PD-1 in the metastatic setting with these subtypes, the lower toxicities associated with anti-PD-1 therapy, the inclusion of these subtypes in adjuvant trials (albeit in small numbers) and the clear RFS benefit in cutaneous melanoma, all make it reasonable to offer adjuvant anti-PD-1 to these patients. In addition, particularly for MM, neoadjuvant therapy is being used increasingly in the clinic. This is largely due to the high morbidity of surgeries needed to achieve adequate margins (eg, pelvic exenteration, abdominal perineal resections, extensive sinus resections), and the high risk of local and distant recurrence despite these surgeries. At this time, there are no systematic data to guide neoadjuvant ipilimumab and nivolumab in MM, although it may have clinical utility, particularly for patients that cannot have a tumor resection without an extremely morbid surgery.

### Targeted Therapies

The identification of genetic alterations and the development of targeted therapies has drastically changed the therapeutic landscape in melanoma. Nearly 50% of cutaneous melanomas harbor *BRAF* mutations and can be successfully targeted with BRAF inhibitors in combination with a downstream MEK inhibitor (34). Combination therapy with BRAF and MEK inhibitors has led to significantly improved OS and progression-free survival (PFS) in advanced cutaneous melanoma (35,36). Unfortunately, the distinct mutational landscapes of acral and MM has limited the use of targeted therapies such BRAF and MEK inhibitors in these populations of patients. Only 3-11% of MM and 15%-20% of AM harbor activating *BRAF* mutations (36,37) thus limiting the number of patients that can benefit. Of note, the rarity of these tumors prevented subgroup analysis in earlier trials evaluating BRAF/MEK inhibitors. As a result, the efficacy of these agents specifically in mucosal and AM remains poorly characterized. In a retrospective analysis of 28 acral and 12 patients with mucosal *BRAF*-mutant melanoma treated with BRAF inhibitors, objective response rate (ORR) of BRAF inhibitors were 38.1% and 20%, respectively (38). A small study from Japan suggested that patients with *BRAF* V600 mutated AM/MM had similar response rates to those with CM (64.3% vs 76.5%) (39). These retrospective studies demonstrated that patients with *BRAF* mutated acral or MM can still derive clinical benefit from BRAF inhibitors (40,41). Therefore, combination therapy with BRAF and MEK inhibitors should be considered for patients with *BRAF* mutations and resected stage III/IV (as adjuvant therapy) or metastatic acral/MM.

Acral and MM demonstrate higher frequencies of genetic aberrations in *KIT*, a receptor tyrosine kinase, than cutaneous melanomas. Activating mutations in *KIT* can be found in up to 36% of AM and 39% of MM, which is considerably more than in melanomas arising from chronically sun damaged skin (1%-7%) or intermittent sun-damaged skin (rare to never) (42). Within MM, *KIT* mutations are much more common in melanomas affecting the vulvovaginal tract compared with sinonasal melanomas (43). Several phase II trials evaluating *KIT* inhibitors in *KIT* mutant melanomas have been conducted with modest activity. Imatinib is the most wel- studied *KIT* inhibitor and is currently recommended for the treatment of *KIT*-mutated melanoma (44). The 3 phase II trials of imatinib showed that responses were highly variable, suggesting that a limited subset of patients with *KIT* mutations (although not amplifications) could derive clinical benefit from *KIT* inhibition (45-47). In these trials, mutations particularly in exons 11 (ORR 45.4%) and 13 (ORR 33.3%) were found to be associated with response. Median PFS was 12-14.8 weeks and median OS was 46.3-56 weeks. Notably, patients with exclusively *KIT* amplifications (without concurrent mutations) did not respond to treatment (45). Moreover, Carvajal et al observed that all 6 responses occurred in tumors with L576P or K642E mutations (47). They found no significant association between clinical melanoma subtype and response (47). Although some patients had rapid and meaningful responses, most patients ultimately progressed within 1-2 years. Due to the wide distribution of mutations in *KIT* and the overall infrequency in which *KIT* mutations are observed, it remains a challenge to conduct any large-scale clinical trials in this population. Two phase II studies suggested that outcomes from nilotinib, another *KIT* inhibitor, appear broadly similar to those observed with imatinib in patients not previously treated with *KIT* inhibition, but generally does not reverse imatinib resistance (48,49). Future directions will likely aim to identify more selective molecular criteria for patients to be treated with *KIT* inhibition, which will require distinguishing driver from passenger alterations of *KIT*. Additionally, targeted therapy with *KIT* inhibitors could be considered as an adjuvant option for patients with resected acral or MM, although it is unlikely that a randomized trial could evaluate this given the rarity of these tumors.

Alterations in MAPK, PI3K/AKT/PTEN, TERT, WNT, and CDK4/CDKN2A are frequently seen in AM (50,51). While these mutations represent possible actionable targets, there are limited targeted therapies available with studies evaluating PI3K/Akt/mTOR inhibitors, CDK inhibitors, and MDM2/p53 inhibitors currently ongoing (52-54). In MM, driver mutations in NRAS, NF1, CTNNB1 and amplifications in CDK4 are commonly reported (55). Inhibition of downstream targets of NF1 (eg, MEK inhibitors) may serve as a potential treatment strategy; however, studies of this approach have not yet been extensively pursued (56). ERBB2 amplifications, which are present in around 3% of acral and MM, are another potential amenable target. In one case report, an ERBB2 amplified patient with AM who had been resistant to checkpoint inhibition obtained a complete response to trastuzumab emtansine (57). Finally, melanomas, including MM, may also rarely have NTRK3 fusions, and in these unusual cases benefit from TRK inhibitors (58).

### Immunotherapy for Advanced Disease

Immune checkpoint inhibitors (ICIs) against cytotoxic T-lymphocyte antigen-4 (CTLA-4) and programmed death-1 (PD-1) have revolutionized the management of cutaneous melanoma. Ipilimumab, a monoclonal antibody against CTLA-4, was the first ICI approved by the FDA in 2011. In 2014, the FDA approved nivolumab and pembrolizumab, 2 mAbs targeting PD-1, as first-line agents for metastatic melanoma (59). Several trials have demonstrated durable response rates of 30%-40% with either pembrolizumab or nivolumab in treatment naïve and previously treated patients with metastatic melanoma (60-62). Unfortunately, due to the rarity of acral and MM, earlier trials did not report response rates separately for these subtypes (63). As a result, there is more limited knowledge regarding the efficacy of ICIs in these subtypes. The distinct mutational landscapes and biology of these specific subtypes would suggest that they may not share the efficacy profiles seen with cutaneous melanoma.

### Immunotherapy in AM

Immune checkpoint blockade appears to have clinical efficacy in AM based on smaller retrospective studies and subgroup analysis in clinical trials. In a retrospective study, Shoushtari et al reported an ORR of 32% [95% CI, 15%-54%] in 25 patients with AM treated with either pembrolizumab or nivolumab, similar or slightly lower than that seen in cutaneous melanoma (63). Treatment was generally well tolerated with comparable occurrences of irAEs to patients with cutaneous melanomas. Another retrospective study showed that patients with AM treated with checkpoint inhibitors had median OS of 17 months, significantly shorter than that of patients with cutaneous melanomas (median OS of 46 months *P* = .047) (62). Moreover, a recent retrospective study of 38 patients with AM treated with anti-PD-1 therapy demonstrated worse ORR (21%) compared with reported data for non-acral patients with a median PFS of 3.6 months and median OS of 25.7 months (64). In a phase II study, Namikawa et al evaluated nivolumab in combination with ipilimumab in 7 patients with AM and reported an ORR of 42.9% (3 of 7, 95% CI, 9.9-81.6), which is comparable to response rates in non-AM (65). The safety of the combination regimen was also comparable to those reported in previous studies (frequency of grade III or IV AEs 77% vs 68.7% in CheckMate 067) (63,66). Despite the worse outcomes compared to those seen in cutaneous melanoma, these studies still support the use of ICIs in AM since survival outcomes are improved over those reported prior to the advent of ICIs.

Of note, there is evidence to suggest that the efficacy of PD-1 blockade in AM may be influenced by ethnicity. A retrospective study of 193 Japanese patients with AM treated with PD-1 blockade found ORRs of 21.1% in the palm and sole group and 8.6% in the nail apparatus group and median OS of 22.3 and 12.8 months, respectively (67). Another study of Chinese patients treated with pembrolizumab provided an overall ORR of 15.8% [95% CI, 6%-31.3%] and a median OS of 12.1 months (95% CI, 10%-25.3%) (68). Notably, these response rates appear possibly lower than those conducted in Western countries. The differences in the immune system between Caucasian and Asian populations may partly account for the reduced efficacy of anti-PD-1 antibodies in the Asian population (67). Despite the potentially reduced efficacy of ICIs in Asian populations compared to Caucasian populations, they still provide antitumor activity and should be considered in this specific subset of patients. Further research is needed to better understand the efficacy of ICIs in AM with consideration given to ethnicity and anatomic location.

### Immunotherapy in MM

Immunotherapy has also demonstrated clinical efficacy in MM, although the data have many of the same constraints seen with AM. A retrospective study by Shoushtari et al identified 35 patients with MM treated with either pembrolizumab or nivolumab and reported an ORR of 23% [95% CI, 15%-54%] with a median PFS of 3.9 months (63). Treatment was well tolerated with very few patients having to discontinue due to toxicity. Another retrospective study involving 59 patients with MM had comparable findings with an ORR of 15.2%, median PFS of 3 months, and OS of 20.1 months (64). Of note, the authors found that elevated serum lactate dehydrogenase was associated with shorter OS (HR, 0.2; 95% CI, 0.08-0.53; *P* = .001). Findings from these studies demonstrate lower response rates to anti-PD-1 therapy in MM, consistent with other reports investigating MM with ORR 0%-23% (68-70). Ethnicity may also influence response to ICI therapy in MM with lower response rates to ICIs being reported in Asian patients with MM. For instance, in one study of 15 Chinese patients treated with pembrolizumab, the ORR was 13.3% (95% CI, 1.7%-40.5%) (68).

Given the lower clinical efficacy of anti-PD-1 in MM, there are ongoing efforts to explore other treatment strategies including combination therapy and radiotherapy. The efficacy of PD-1 blockade in MM was evaluated in a pooled analysis involving 86 patients with advanced MM treated with nivolumab monotherapy and 35 patients with MM treated with combination ipilimumab and nivolumab (69). For patients who received nivolumab monotherapy, ORR was 23.3% [95% CI, 14.8%-33.6%] in patients with MM and 40.9% [95% CI, 37.1%-44.7%] in patients with cutaneous melanoma (69). Among patients who received combination nivolumab and ipilimumab, response rates were higher, with ORR of 37.1% and 60.4% for mucosal and cutaneous melanoma subtypes, respectively (69). This analysis provides support for the efficacy and safety of PD-1 blockade in MM, and in addition, demonstrates the potential greater efficacy found with combination therapy. However, in a retrospective, multicenter study of 329 cases of MM in Japanese patients, there were no significant differences between anti-PD-1 monotherapy and combination therapy (PD1 + CTLA4) with regards to ORR (26% versus 29%; *P* = .26), PFS (median PFS 5.9 months vs 6.8 months; *P* = .55) or OS (median OS 20.4 months versus 20.1 months; *P* = .55) (71). Another strategy that has been explored to improve the low efficacy of ICIs in MM is ICI therapy in combination with radiotherapy. A multi-institutional retrospective study of 225 Japanese patients with MM evaluated anti-PD-1 monotherapy or anti-PD-1 + anti-CTLA-4 combination therapy with or without radiotherapy and found no survival benefit with RT in either ICI regimen (72). In the PD-1 cohort, ORR was 26% in PD1 alone versus 27% in PD1 + RT (*P* > .99), and similarly, in the combination therapy cohort, ORR was 28% in PD1+CTLA4 vs 25% in PD1+CTLA4+RT (*P* = .62) (72).

In summary, immunotherapy is a promising treatment option for patients with advanced acral and MM. Combination ipilimumab and nivolumab likely has increased clinical benefit compared with single agent anti-PD-1, although results (particularly in MM and in Asian populations) have varied across trials. Although efficacy is lower than in cutaneous melanoma, it offers notable clinical benefit for patients who have otherwise limited therapeutic options. Combination immunotherapy has been associated with higher response rates in these rare subtypes and should be considered in those that can better tolerate the potential greater toxicities (Table 1).

**Table 1.** Summary of immunotherapy studies in acral and mucosal metastatic melanoma.

Melanoma subtype	Study^a^	Patients (*N*)	Treatment (line of therapy)	Response rate (%)	Median survival notes (months)
Acral	Shoushtari et al (63)	25	Anti-PD-1 (mixed)	32	PFS: 4.1 OS: 31.7
	Klemen et al (62)	22	Anti-CTLA-4, anti-PD-1, and/or anti-PD-L1 (mixed)	Not reported	PFS: not reported OS: 17
	Ogata et al (64)	38	Anti-PD-1 (mixed)	21	PFS: 3.6 OS: 21
	*Namikawa et al* (65)	7	Ipilimumab + Nivolumab (1^st^ line)	42.9	PFS: not reported OS: not reported
	Nakamura et al (67)	193	Anti-PD-1 (mixed)	16.6	PFS: 3.5 OS: 18.1
	*Si et al* (68)	39	Pembrolizumab (2^nd^ line)	15.8	PFS: 2.8 months (aggregate) OS: 12.1 (aggregate)
Mucosal	Shoushtari et al (63)	35	Anti-PD-1 (mixed)	23	PFS: 3.9 OS: not reported
	Klemen et al (62)	38	Anti-CTLA-4, anti-PD-1, and/or anti-PD-L1 (mixed)	Not reported	PFS: not reported OS: 18
	Ogata et al (64)	59	Anti-PD-1 (mixed)	15.2	PFS: 3 OS: 20.1
	*Namikawa et al* (65)	12	Ipilimumab + Nivolumab (1st line)	33.3	PFS: not reported OS: not reported
	*Si et al* (68)	15	Pembrolizumab (2nd line)	12.5	PFS: 2.8 months (aggregate) OS: 12.1 (aggregate)
	D’Angelo et al (69)	86	Anti-PD-1 (mixed)	23.3	PFS: 3 OS: not reported
		35	Ipilimumab + Nivolumab (1st line)	37.1	PFS: 5.9 OS: not reported
	Nakamura et al (71)	263	Anti-PD-1 (1st line)	26	PFS: 5.9 OS: 20.4
		66	Ipilimumab + Nivolumab (1st line)	29	PFS: 6.8 OS: 20.1
	Umeda et al (72)	115	Anti-PD-1 (1st line)	26	PFS: 6.2 OS: 19.2
		42	Anti-PD-1 + radiotherapy (1st line)	27	PFS: 6.8 OS: 23.1
		56	Anti-PD-1 + anti-CTLA-4 + radiotherapy (1st line)	28	PFS: 5.8 OS: 31.7
		12	Anti-PD-1 + anti-CTLA-4 (1st line)	25	PFS: 3.5 OS: 19.8

Italic = trial; all others were retrospective studies.

## Future Directions

Therapies for acral and MM remain limited, especially in comparison to cutaneous melanoma. Targeted therapy may be an adjuvant therapy option for patients with actionable driver mutations (specifically *BRAF* mutations); however, more efforts are needed for targeted therapies in *BRAF* wild type patients, potentially targeting *KIT*, CDK, TERT, and other pathways. Additionally, as the understanding of the biology and mutational landscape of these subtypes progress, targeting of other mutated genes may be pursued in the future.

The lower activity of anti-PD-1 in acral and MM has led to novel combination strategies being explored (30,62-64,68). For example, the combination of the PD-1 antibody toripalimab with the vascular endothelial growth factor inhibitor, axitinib, appears to be a promising treatment option for patients with MM. A phase Ib trial of axitinib in combination with toripalimab in patients with MM showed an improved ORR 51.7%, and median PFS 7.5 months (73). It is not clear whether this combination may be used after progression on either single agent anti-PD-1 or ipilimumab and nivolumab. Another potential therapeutic option that is being actively studied is nemvaleukin, alfa, an engineered interleukin-2 (IL-2) variant. It was recently granted fast-track designation by the FDA for the treatment of patients with MM who have been previously treated with checkpoint inhibitors based on a few responses observed in an earlier phase I study (74). Finally, additional studies are needed to define the role of neoadjuvant and adjuvant therapy, particularly for MM.

## Conclusions

Despite the remarkable advancements in the treatment of cutaneous melanoma, there is a paucity of evidence to guide management in patients with acral and MM. The rarity of these subtypes has made it difficult to conduct large randomized controlled trials of targeted therapies and ICIs that have emerged in recent years. Future directions should aim to better understand the biology of these pathologic subtypes and define more active combination therapy approaches.

## Data Availability

Data sharing is not applicable to this article as no datasets were generated in the writing of this review.
